# E-learning strategies from a bioinformatics postgraduate programme to improve student engagement and completion rate

**DOI:** 10.1093/bioadv/vbac031

**Published:** 2022-05-10

**Authors:** Andrés Garzón, Alejandro Rubio, Antonio J Pérez-Pulido

**Affiliations:** Andalusian Centre for Developmental Biology (CABD, UPO-CSIC-JA), Department of Molecular Biology and Biochemical Engineering, Faculty of Experimental Sciences, University Pablo de Olavide, Seville 41013, Spain

## Abstract

**Motivation:**

E-learning is the standard solution adopted in transnational study programmes for which multiple face-to-face learning places are not an option. Bioinformatics is compatible with e-learning because its resource requirements are low. Online learning, however, is usually associated with high dropout rates because students start from a very low computational level and/or they need support to conduct practical analyses on their own.

**Results:**

In this article, we analyse the academic results of an online bioinformatics educational programme based on learning communities. The programme has been offered by the Spanish Pablo de Olavide University for more than 5 years with a completion rate of close to 90%. Learning bioinformatics requires technical and operational competencies that can only be acquired through a practical methodology. We have thus developed a student-centred and problem-based constructivist learning model; the model uses faculty and peer mentoring to drive individual work and retain students. Regarding our innovative learning model, the recruitment level (i.e. the number of applicants per available places and international origin), the results obtained (i.e. the retention index and learning outcomes) as well as the satisfaction index expressed by students and faculty lead us to regard this programme as a successful strategy for online graduate learning in bioinformatics.

**Availability and implementation:**

All data and results for this article are available in the figures and supplementary files. The current syllabus (Supplementary File S7) and other details of the course are available at: https://www.upo.es/postgrado/Diploma-de-Especializacion-Analisis-Bioinformatico and https://www.upo.es/postgrado/Master-Analisis-Bioinformatico-Avanzado.

**Supplementary information:**

Supplementary data are available at *Bioinformatics Advances* online.

## 1 Introduction

In Biological Sciences, the capacity to generate scientific data grows annually at an exponential rate. A paradigmatic example is the accumulation of information from biological sequences. The emergence of high-throughput sequencing techniques and increasingly cost-effective methodologies result in sequence databases that, at the time of publication of this article, store nearly a billion sequences consisting of trillions of characters (O’Leary *et al.*, 2016; UniProt Consortium, 2019). In fact, the number of available sequences approximately is doubling approximately every 18 months and is now accompanied by other biological data, such as the results of gene expression experiments, which are also growing exponentially (Barrett *et al.*, 2013). This plethora of biological data must be analysed to generate new knowledge that is useful in different branches of science. Molecular biologists are often not adequately trained in the computational analysis of this type of data (Wilson Sayres *et al.*, 2018). Although they are responsible for deriving knowledge from wet lab results, they do not have the necessary skills to handle this volume of information due to the lack of specific training in computational techniques. This capacity for handling and managing data on a massive scale is suited to technically oriented professions such as computer engineers and programmers. But these latter professionals lack the necessary skills to understand the biological problems they work on. It is thus difficult to develop specific, tailor-made solutions adapted to solving today’s biological problems. Multidisciplinary teams are usually created to address this problem and respond to the lack of professionals specifically trained for these tasks. However, this is not always the best solution, due to the lack of comprehensive training in bioinformatics, which has even been recommended to be considered before the undergraduate level (Machluf and Yarden, 2013).

Today, there is a need for professional profiles that combine knowledge and skills in biology, information technology and programming (Cummings and Temple, 2010). This fact had already been recognized when bioinformatics was nascent and millions of sequences had been stored in public databases (Kanehisa and Bork, 2003). Nevertheless, specific university training is still in high demand today (Attwood *et al.*, 2017), so undergraduate and postgraduate academic programmes in this field continue to emerge (Ahmed *et al.*, 2020; Brazas and Ouellette, 2013; Gurwitz *et al.*, 2017; Kulkarni-Kale *et al.*, 2010; Via *et al.*, 2013).

While these degrees are appearing and consolidating in Spanish universities, demands for continuous training cannot be left unaddressed. Researchers carrying out their PhD thesis, postdoctoral stays, and even senior researchers whose projects are beginning to generate quantities of data that are difficult to handle are asking for specialized training in computational techniques that will allow them to further progress in their work. For their part, computer engineers and programmers who work on biological projects also demand specific training enabling them to better understand the biological problems they are tackling and thus to participate more creatively in solving them.

Massive Open Online Courses (MOOCs) are often presented as a solution. However, the lack of guidance and support from teachers leads to course completion rates of only 10% (Jordan, 2015; Kizilcec *et al.*, 2017).

Responding to these demands, in 2015, the Pablo de Olavide University in Seville designed a practical postgraduate continuing education programme in bioinformatics. The course takes place entirely online in an asynchronous way, while conducting a close student follow-up that ensures the training is consolidated and practical. Online learning models, apart from MOOCs, often have low completion rates due to early dropout, mainly because of lack of planning or frustration when confronted with new problems and techniques (Levy, 2007; Sweeney *et al.*, 2008). In this article, however, we will show how these issues are addressed in our programme, achieving a dropout rate below 20%. We describe the programme’s academic and instructional design, the academic results as well as the degree of satisfaction of students and faculty.

## 2 Instructional design

We started this project by analysing the requirements of graduate studies in bioinformatics and concluded that the potential audience would mainly be active researchers in leading fields of biological research, who would be prioritizing their current work and would be seeking a flexible training modality, if possible, without the need to travel. Such a profile, therefore, is highly adapted to online learning. In addition, bioinformatics is one of the few biological disciplines that does not require wet-labs and can be taught with little more than a computer and a good Internet connection. The design of an online programme also offers other advantages, including internationalization, allowing the course to be taken anywhere. In this way, our programme had the potential of covering the demands for bioinformatics graduate courses in Spain as well as in Latin America, since the activities are proposed in an asynchronous way.

Successful e-learning requires highly motivated students and trainees, together with well-prepared high-quality materials (Via *et al.*, 2013). The Pablo de Olavide University has a fairly large number of bioinformatics researchers, many of whom are also university professors. The course’s faculty includes these researchers together with senior faculty experts in e-learning. In the subjects for which it was necessary, researchers’ scientific networks were used to recruit professors from other national and foreign research centres, since the online model also allows the incorporation of international teaching staff.

**Fig. 1.**
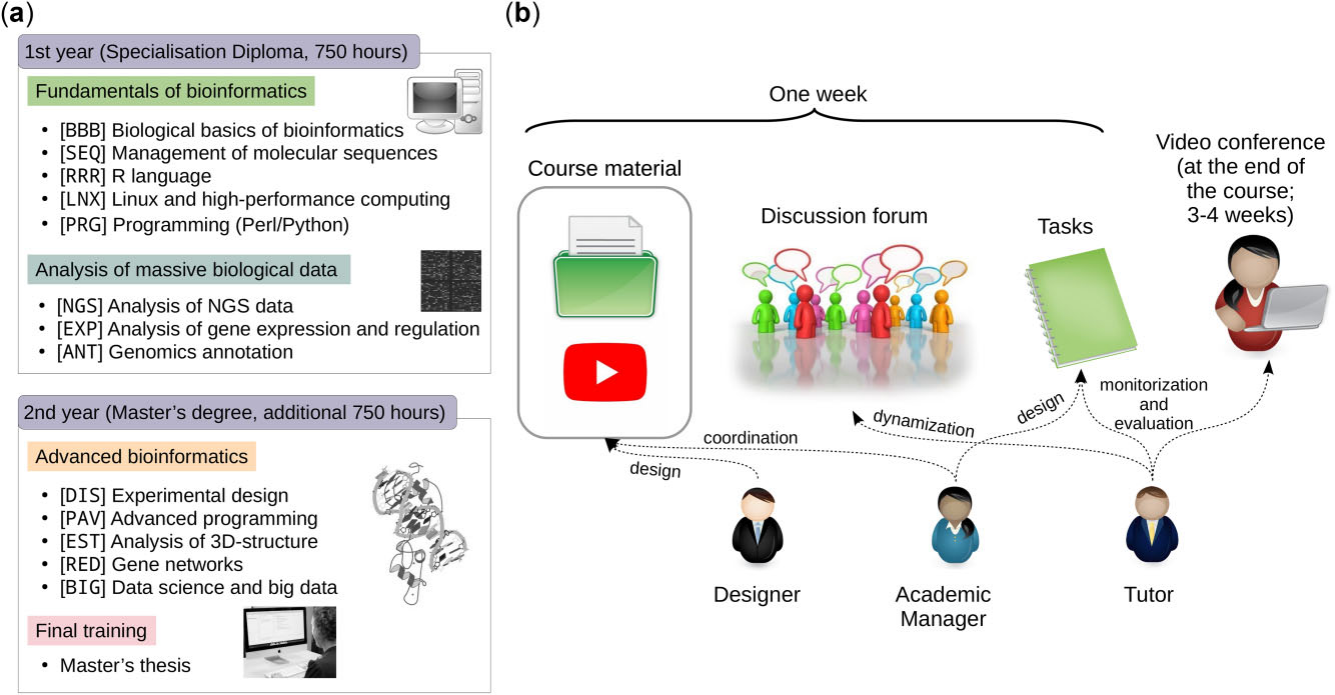
Curriculum and teaching model of the programme. (**a**) Curriculum developed in the postgraduate course. The list of subjects by year is shown grouped by modules (the acronym of each subject is shown in brackets). The first module includes fundamentals in bioinformatics and technical subjects, covering sequence alignments, databases, phylogenomics and programming languages. The second module is oriented toward massive data analysis. The programming subject started with Perl language, but it was changed to Python after the fourth edition. In the same way, Linux started after the third edition, and Genomics annotation was initially divided into two subjects: Structural and Functional Annotation. The first year can be taken independently to obtain a specialization diploma. The second year tackles more specialized tasks used in bioinformatics, as well as novel topics such as Data Science, and finishes with a Master’s thesis. When a student passes both courses, he/she obtains the master’s degree. (**b**) Learning model including different teaching roles. The three teaching staff figures are shown below, with arrows describing their main roles. The three main blocks of activities programmed by week for students are grouped above. The final interview is performed by the tutor via video conference with each student

Initially, the basic knowledge and computational skills required by a graduate in experimental sciences or computer science to be able to solve biological problems using bioinformatics were identified, starting from a level of zero knowledge (Fig. 1a). From there, curricular subjects were proposed and then experts in each of them were sought who could be in charge of the content design and coordination of each subject.

The future students’ professional profiles led us to believe in an online programme design since these potential participants have a great capacity for self-learning and are accustomed to obtaining and digesting information via their own means. Nevertheless, such profiles also present high dropout risks as the course would be competing with the innumerable obligations imposed by a life dedicated to scientific research. This made it necessary to direct the instructional design toward combatting dropout risks.

Early online course abandonment is often due to the following widely identified factors: boredom, feelings of loneliness, the learning platform’s technical complexity, lack of support for technical problems, lack of help with questions on certain concepts, lagging response times, difficulty in self-managing the pace of work and accumulation of work near due-dates (Bawa, 2016; Goda *et al.*, 2015). Therefore, we designed the learning strategy focusing on maximizing student retention. One of our major innovations was the close accompaniment and support given to students.

Below, we summarize the main features, grouping them into a series of 10 strategies to facilitate their inclusion when planning a new course.

*I. Part-time curriculum design.* A master’s degree in Spain includes a minimum of 60 ECTS credits (European Credit Transfer and Accumulation System), i.e. the equivalent of 1 year of full-time work or about 1500–1800 h of student work (Publications Office of the European Union, 2017). Given the applicants’ professional profile, we decided to split the programme into two courses with 30 ECTS credits each, so that they could be taken on a part-time basis. The first academic year (from October to June) addresses the basic disciplines and can be taken as an intermediate independent degree by students who do not need to study further (Fig. 1a). The second year is mainly dedicated to certain specific subjects in bioinformatics and aims at improving programming competencies, finishing with a practical, hands-on master’s thesis, usually in a research project supported by one of the course teachers.

*II. Establishment of different teaching roles.* To fulfil the requirements of the educational model, the teaching staff can assume three types of independent roles in each subject: designer, academic manager, and tutor (Fig. 1b). The designer is a senior teacher who builds and updates the subject’s materials and establishes the instructional design and assessments. The academic manager directs a subject’s academic development and oversees the evaluation design as well as the tutors’ coordination. Finally, the tutor is probably the most relevant figure in this student-centred teaching scheme, since he/she is in charge of the follow-up and evaluation of a small group of students (20 maximum), accompanying them during a subject’s duration of study. Tutors are active researchers with expertise in the corresponding subject. They must be proactively attentive to students at risk of dropout, those who are not achieving the learning objectives, and those who are not participating in a constant way. Consequently, this figure constitutes one of the main instruments against dropout since the student does not feel abandoned at any time of the course. When a subject has ended, tutors write reports on each student in their group, which is forwarded to the corresponding tutor of the following subject. The latter can thus have an initial idea of the strengths and weaknesses in the student group. In fact, interactions between teachers are continuous during the course.

*III. Modularity*. Subjects are limited in time, usually 1 month in duration, so students only work on one subject at a time (Fig. 1b). Each subject presents contents and tasks on a weekly basis, so each week is an independent work unit with deliverables at the end of the period. This prevents the student from accumulating undelivered assignments and helps to avoid procrastination.

*IV. Virtual campus maintained by the university*. A robust and well-attended Learning Management System (LMS) plays a key role in supporting the learning model. The course makes use of a robust digital platform maintained by the University. Most teachers are accustomed and well-trained in the management of this platform, which includes different learning tools such as blogs, wiki pages, and discussion forums, as well as the standardization of resources. For the sake of uniformity, the course has a single virtual space for all subjects, which is standardized by the LMS manager, who also organizes the discussion forums.

*V. Uniform structure of the virtual space for all subjects.* All subjects have a homogeneous design, which avoids students wasting time learning a different working methodology for each one. As indicated above, all subjects are organized within the same LMS space, with shared resources such as common instructions, grading book or the software installation instructions. This virtual space is maintained by an LMS manager who ensures that everything is well presented, and all subjects follow the common standards.

*VI. Homogeneous work scheme.* When starting a new subject, the student is provided with an outline that always maintains the same working model. The weekly scheme consists of theoretical contents mainly configured as SCORM compliant HTML pages, one or two explanatory videos, a guided practical application and a final evaluation, mainly consisting of a practical exercise (Table 1). This model ensures that students acquire operational skills by the end of each weekly unit.

**Table 1.** Activities commonly used by the different subjects of the course, together with their frequency and some real examples

Activity	Timing	Description	Examples
Discussion forum	2–3 times a week	Discussions proposed by the teachers to deepen the knowledge and applications of a subject and build new knowledge in a collaborative fashion	Strategies to solve a practical problem with R, Linux commands or Python or how to analyse the secondary structure of a protein
Guided exercise	Several per subject	Step-by-step tutorial on how to perform a task followed by a similar exercise to fulfil by every student supported by forum discussions	Search for sequence motifs in a genome using Linux commands or a Python script
Weekly practical task	Once a week	Evaluation exercise with a case study	Carry out the assembly of a genome or a variant discovery analysis from high-throughput sequencing
Collaborative wiki page	Sometimes	Collaborative information search and creation of a content page	Complete the functional annotation of a protein using different sources (groups of 5–8 students)
Final task	Once per subject	Final exercise of the subject which can encompass everything learnt in the course; this is also the only exercise to pass the subject in a second attempt	Writing a programme to combine information coming from entries of two different databases, or complete the annotation of a bacterial genome including both protein-coding and non-coding genes
Final video conference	Once per subject	Short individual meeting in which one of the practical tasks is reviewed and the subject is passed if authorship of the exercise is shown	How did you perform the RNA data analysis?

*VII. Weekly discussion forum.* The most complex theoretical concepts are discussed over the discussion forums, as well as the difficulties encountered during the tasks. The teaching staff moderates these debates, introduces new questions, challenges and resolves queries. Forums are a key feature of our problem-based constructivist learning model, substituting classroom interaction and allowing the construction of knowledge over a learning community. Participation in forums is evaluated and requires continued quality participation. It constitutes 20% of the total grade in each subject. All of this ensures a high student participation (more than 100 messages a week), with a continuous coordination from the tutors in each subject.

*VIII. Digital materials.* The study materials are provided in SCORM standardized web format (standard HTML documents and videos) and are not delivered in printable format (PDF or similar). This increases the time that students are connected to the LMS and, therefore, their disposition to communicate with their peers and faculty. In addition, students can download and install, at the start of the course, a virtual machine with GNU/Linux OS in which all the software they need is pre-installed. It saves students the chore of installing specific tools, libraries, and requirements for each subject, and ensures that practical examples and evaluation exercises are going to work. This virtual machine is the central tool of the subject on Linux, together with access to a high-performance computing machine which is maintained by our university and is also used in other later subjects of the massive analysis module.

*IX. Continuous evaluation with weekly activities and a final test.* The evaluation deadlines are strict, thus guaranteeing that all students take the same content at the same time. This facilitates interactions over the discussion forums. The model focuses on the application of practical knowledge to solve problems, rather than theoretical examinations. When a subject ends, the tutor conducts a 5–10 min interview to validate the authorship of the student’s deliverables, which is the only synchronous activity of the course.

*X. Quality management system.* Student satisfaction surveys are carried out at the end of each subject, and results are used as a tool for improving the quality of the course. At the end of every course, a general satisfaction survey is also carried out among students and teachers. This is in addition to the impressions gathered by teachers in the discussion forums and in the final video conferences, where students sometimes make suggestions for improvement. A quality report is generated with all the collected information, which is used to present an improvement plan for each subject, as well as for the course as a whole, to be implemented in the next course edition (Supplementary File S1). When the next course ends, the subject academic manager evaluates the results of the improvement plan. The course also has rules regarding the management and resolution of academic incidents by students and teachers.

The course is a non-official master’s degree that differs from the official one in that it does not give the right to defend a doctoral thesis. It is mainly aimed at recent graduates, PhD students and professionals, both in experimental sciences and computer engineering, allowing them to acquire practical skills to analyse their own data, as well as to obtain a university certificate upon completion. The admission criteria are based on three fundamental points: degree and grades according to the academic transcript (66.66%), current professional situation and previous knowledge of bioinformatics (33.33%). As an example, recent graduates who are working on a thesis or have a research contract, as well as professionals with a more extensive curriculum, do not usually have problems in gaining access to the course.

## 3 Methods for measuring the e-learning programme

The results presented in this article correspond to the first five editions of the first annual cycle of the master’s degree (academic years 2015–2016 to 2019–2020), as this is the period for which we have the most complete time series of data.

### 3.1 Analysis of enrolment and final grades

Objective enrolment and performance indicators were collected from the results of the course. The selected objective indicators were as follow:

- Admission demand. Number of applications for admission. It was compared with the number of places offered each academic year.
- Admission profile. Number of Bsc, Msc and PhD students enrolled.
- Dropout rate. Percentage of initial students who stopped participating in the subjects and stopped taking the evaluations before the end of the course, including cancellations of enrolments with the course already underway.
- Yield rate. Average share of credits passed of the total number of ECTS credit enrolments.
- Average grade. Average value of the grades obtained by students who completed the course.

### 3.2 Analysis of satisfaction

Satisfaction surveys were conducted to analyse the subjective perceptions of students and teachers. Two surveys were carried out for the student satisfaction indicators, one at the end of each subject, and another at the end of the complete programme.

Questions asked at the end of each subject are available in Supplementary File S2. The students were asked to give their degree of agreement with every statement from 1 (strongly disagree) to 5 (strongly agree).

Questions asked at the end of the course are available in Supplementary File S3. The students were asked to give their degree of agreement with each statement from 1 (strongly disagree) to 10 (strongly agree).

To obtain indicators of teacher satisfaction, a survey was conducted at the end of the course. Questions asked at the end of the course are available in Supplementary File S4. Teachers were asked to give their degree of agreement with each statement from 1 (strongly disagree) to 10 (strongly agree).

Results were collected the next week after finishing every subject, and the survey was completed by more than half of students (more than 30 students on average; see Fig. 3a for details).

### 3.3 Analysis of acquired skills

A survey was sent to students from all finished editions of the course. It was open for 1 week, and results were then analysed. The survey’s questions and answers received are available in Supplementary File S6.

## 4 Results

### 4.1 Admission and performance indicators show a low dropout rate

**Fig. 2.**
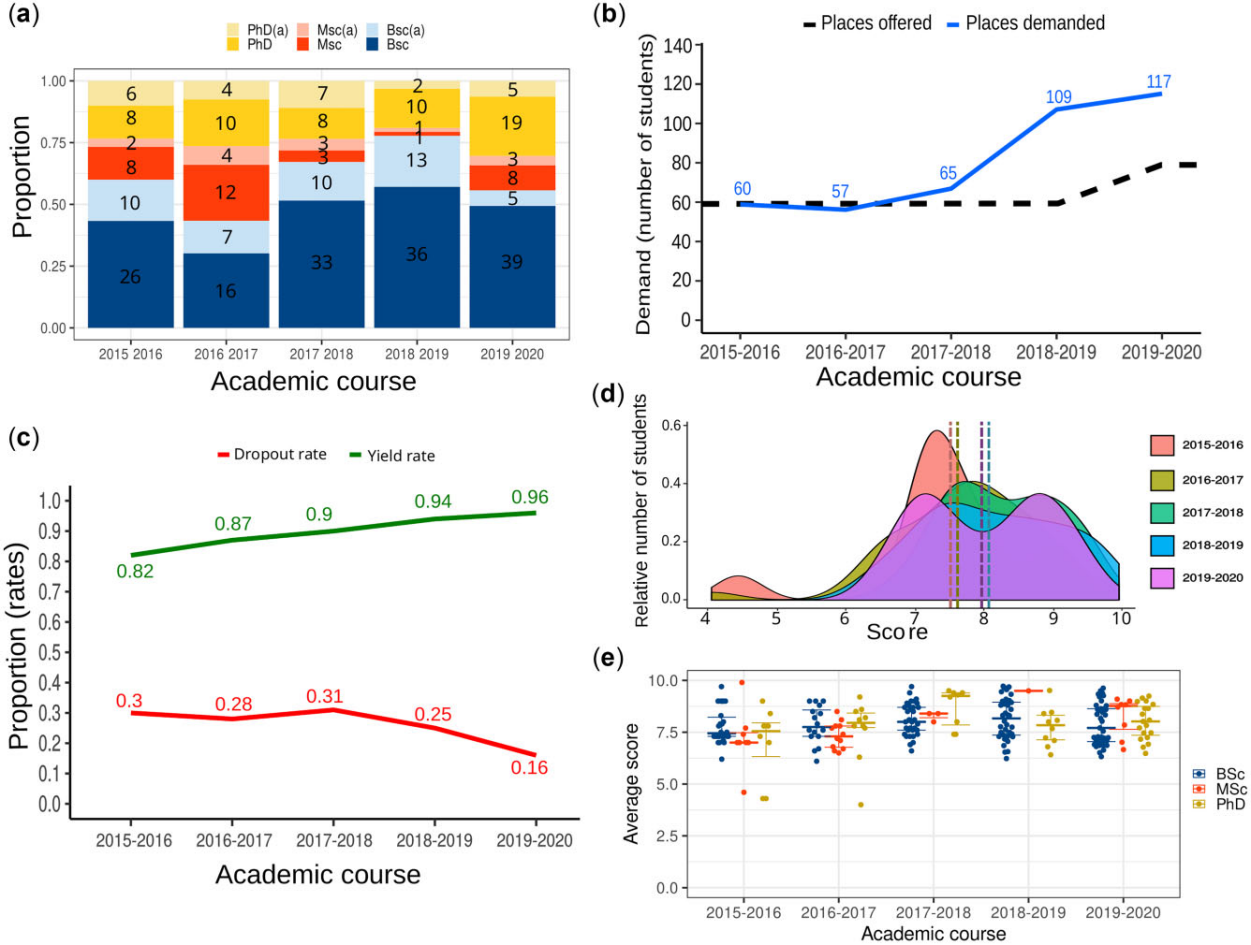
Programme enrolment and performance indicators. (**a**) Yearly evolution of student enrolment distinguished per academic profile. The lighter colours show students who abandoned the course (a). The bars show the proportion of total students, and the absolute number is also labelled by group. (**b**) Yearly evolution of the course demand, where the dashed line shows the vacancies. (**c**) Yearly evolution of both dropout and yield rates. (**d**) Distribution of final grades by edition, (**e**) and grades distinguished per student profile. Grade scale ranges from 0 (fail) to 10 (outstanding)

Enrolment in the programme usually opens about 4 months before it starts in October, and we receive applications from all over the world, mainly from Spain and Latin America. During the five past editions, we have been able to observe a recurring admission profile, with a number of Bsc students similar to the sum of both Msc and PhD (Fig. 2a). During the first four editions, admissions took place following the order of receipt of the applications. But due to the incremental demand, we increased the number of places from 60 to 80 (Fig. 2b), and the received requests are now evaluated, mainly according to academic record and professional profile. Thus, the admission process was completed around September during the first years, but it closed in July for the 2019–2020 edition, demand being around double the number of available places. In the first 5 editions, we had students living in 11 different countries and 38 from the 50 Spanish provinces (Supplementary File S5).

As mentioned previously, student dropout is a major problem in e-learning. To assess whether the learning model implemented succeeded in reducing the dropout rate throughout the different editions of the course, we calculated this value at the end of each edition, together with other performance indicators (see Section 3.1). Thus, the dropout rate for the course started at 30%, a percentage that has been decreasing to 16% in the last edition of the course (Fig. 2c).

In addition to the number of students who abandon the course, another success index of a course is the yield rate, that is, the proportion of credits passed in relation to those evaluated. It ranged from 0.82 to 0.96, increasing in the two last editions, together with an additional reduction in the dropout rate. This further achievement could be explained by the combination of the results of our continuous improvement plan and the selection of student due to the increased demand for enrolment. In addition, it is worth noting that the last 3 months of the last edition analysed coincided with the confinement of the pandemic by coronavirus disease 2019 (COVID-19), which may also have implied a greater dedication to the course (Gonzalez *et al.*, 2020). The final grades obtained accounted for an average of around 7.5 during the first 2 years, increasing to 8 in the following editions (Fig. 2d). It is important to note that these grades have not depended on student profiles (Fig. 2e).

### 4.2 Students’ satisfaction surveys by subject show high satisfaction and support continuous improvement

**Fig. 3.**
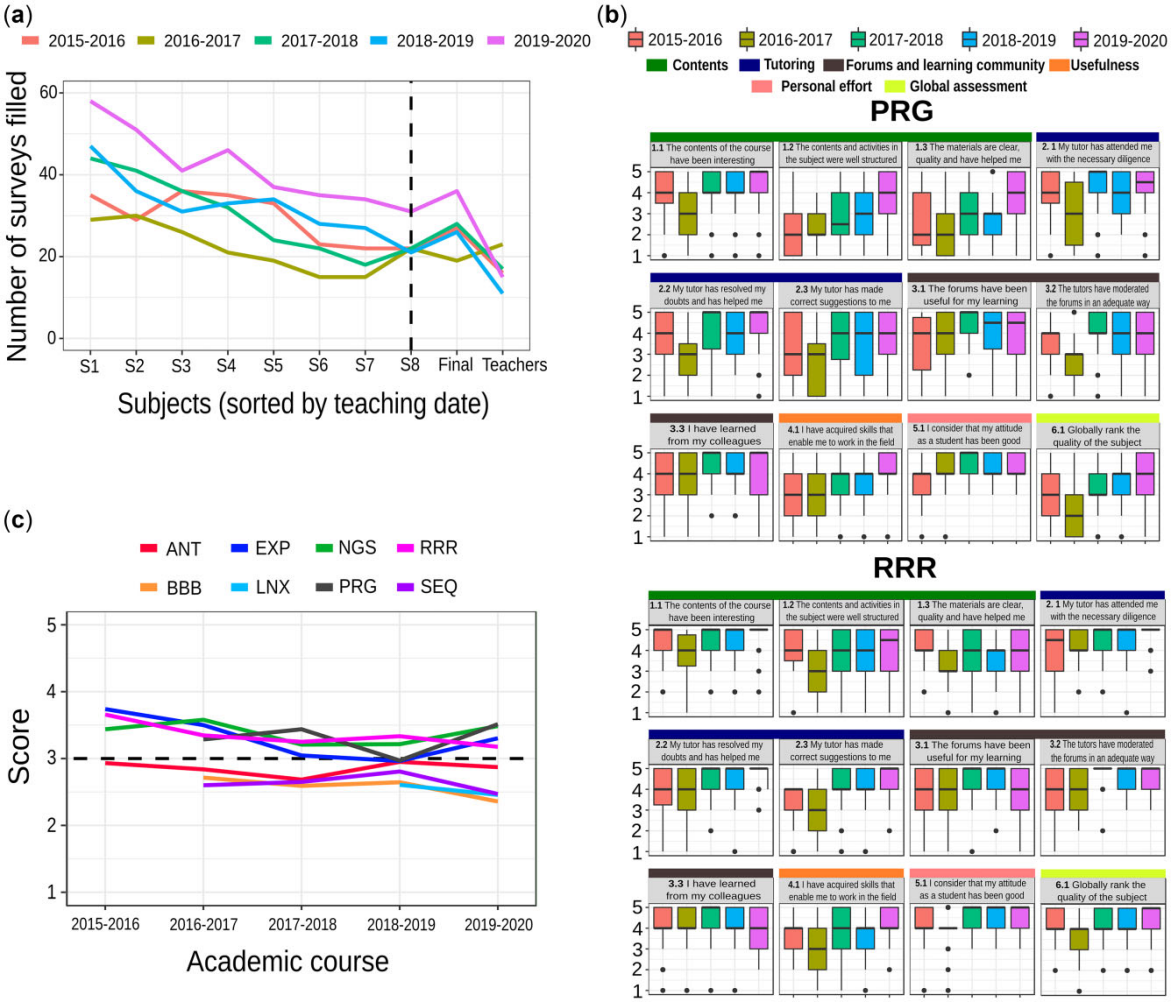
Surveys by subject and edition. (**a**) Number of surveys answered by subject and edition, in addition to the final survey for both students and teachers from the dashed line on. (**b**) Yearly evolution of the mean scores of the satisfaction index obtained for two subjects: Programming language Perl/Python (PRG), and R (RRR). The results obtained from all the programme subjects are available in Supplementary Fig. S1. (**c**) Survey result for the question ‘Daily time dedicated to the subject’, where 3 is the expected time linked to 25 credits, less time is <3 and more time is >3. This question was added to the surveys during the first edition and is lacking for the subjects BBB, SEQ and PRG, as well as the recent subject LNX. The total number of students was around 60 during the first three editions, and 79 in the last edition. In both (c) and (d), the scale ranges from 1 (strongly disagree) to 5 (strongly agree)

To identify points of improvement and to measure students’ levels of satisfaction, a survey is conducted upon completing each subject. This survey includes questions that fall into six main blocks: contents, tutoring, discussion forums, usefulness, personal effort and global assessment. The surveys are proposed to students at the end of each of the eight subjects. They are answered on average by 31 students (48% of them), and participation is expected to be lower as the course progresses (Fig. 3a).

The mean general satisfaction score has been close to 4 out of 5 and was maintained at around 4.3 in the fifth edition. The highest scores are usually obtained in the contents and tutoring blocks, which is significant since tutoring is one of the key valuable features of this programme. The results for subjects related to programming languages, two of the most challenging subjects for students with a scientific profile, present a generally upward trend (Fig. 3b). The latter suggests that past results have improved thanks to the course’s improvement plan, as shown in the perceptions of the structure of both the course contents and activities (Question 1.2), or the perceptions of the usefulness of the acquired skills (Question 4.1). The latter have increased over time, thus fulfilling the aims of any bioinformatics course.

**Fig. 4.**
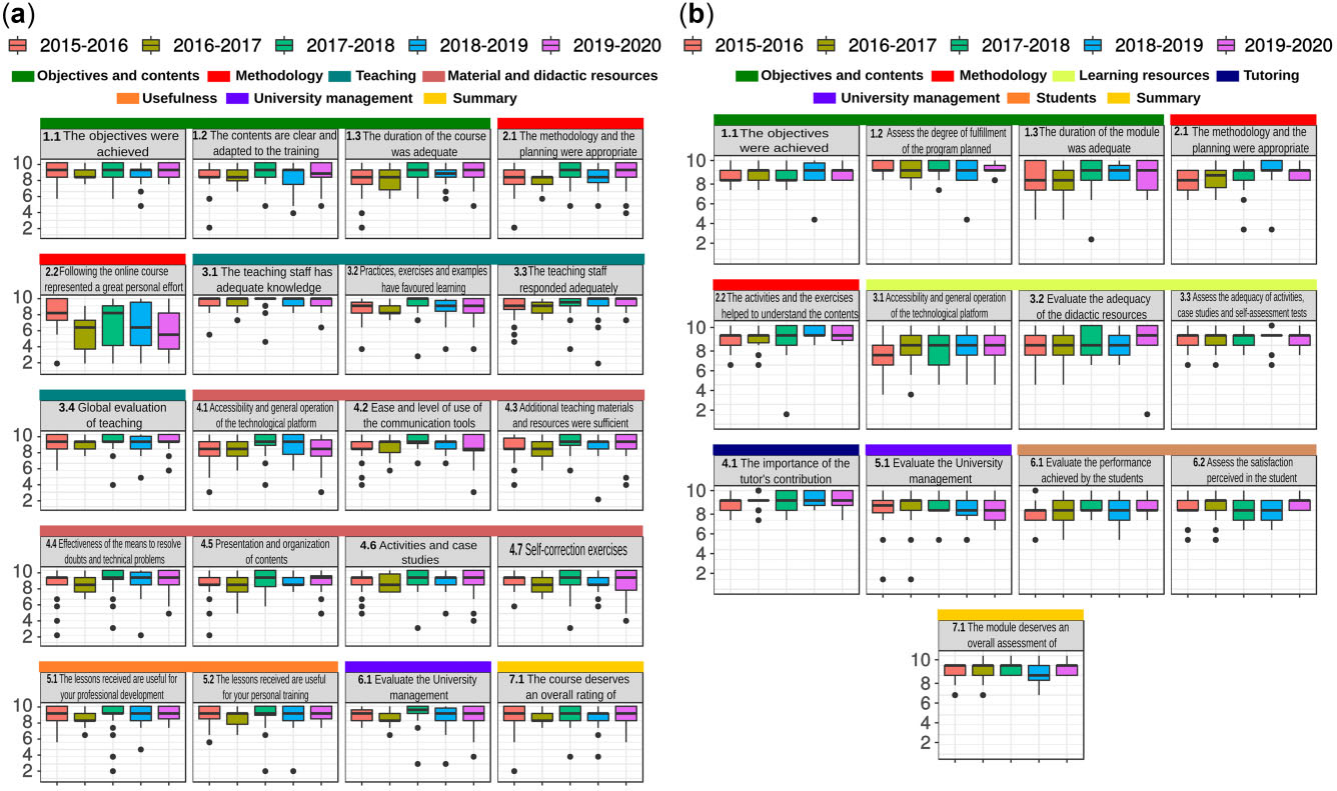
Yearly evolution of scores for the satisfaction index. (**a**) Students and (**b**) teachers. The scale ranges from 1 (strongly disagree) to 10 (strongly agree)

Discussion forums require a more detailed analysis. They are used to establish cooperative learning communities, where knowledge is created. They are therefore evaluated and represent 20% of the total subject score. The survey comments always include some student complaints about the time they must dedicate to reading and writing in forums. However, when finishing a subject, they attribute high scores to items related to tutoring and forum moderation, and they highlight the utility of forums for their learning, together with what they learn from peers there (Fig. 3b). Thus, the score is now higher than in the initial years, in accordance with the positive experience reported by course teachers regarding this task (see Fig. 4b below).

On the other hand, the survey results reflect a certain number of aspects that need to be improved in some subjects. One of these is related to the tutor’s proactivity (Question 2.3). The proposed learning model requires that tutors monitor and supervise their corresponding student group, not only solving the points raised but also enquiring about possible weaknesses or specific support needs. Currently, the programme includes 23 different tutors; they are coordinated both within the subject by the teacher in charge, and across the course via global meetings which address general issues. The need for proactivity has been conveyed to tutors and has been improved thanks to the specific quality plans deployed along the academic years; it is especially noticeable in the item related to the recommendations given by tutors to the students. Proactivity improvements are reflected in the subject surveys along the different editions. But it still needs to increase in programming language.

Another noteworthy item is the student’s dedication time. A score of 1 for this item means that the student has dedicated much less time than the expected 25 h a week. A score of 5 means that the student dedicated much more time, and a score of 3 means that the student dedicated approximately the 25 h expected. The value for this item across all subjects has ranged between 3.75 and 2.5, but it has tended toward 3. The latter suggests that the dedication time is well organized by both students and teachers, achieving a balanced workload (Fig. 3c). This dedication can be also checked by tutors through LMS’s monitoring tools.

### 4.3 Students’ and teachers’ overall course satisfaction help to progressively improve the programme

Per-subject surveys are useful to evaluate each subject and to prepare the yearly improvement plan. But in this course, standardization plays a key role in keeping the students connected. Thus, a general students’ satisfaction survey is conducted after finishing the first annual cycle. Despite being the ninth survey presented to students, it was nevertheless completed by more than one-third of them (Fig. 3a), providing us with an index of global satisfaction (Fig. 4a). The average mark given by the students for the course as a whole has always been above 8 out of 10 and is currently close to 9. In this case, the items are divided into seven blocks: objectives and contents, methodology, teaching, resources, usefulness, university administration and summary. The highest scored item has always been related to the teaching staff’s knowledge about the course contents, together with the proposed practice and exercises (Questions 3.1 and 3.2). This latter result is noteworthy since the course aims at providing highly practical training in bioinformatics analysis. In addition, the scores show a positive trend over the successive course editions, which could be related to both the faculty’s accumulated experience, and the students’ higher profiles.

Again, when students are asked whether the online course represents a greater personal effort than a traditional face-to-face or distance course (Question 2.2), they first answered that the effort was greater, but the yearly trend has reached a value of 5, indicating that the perceived effort is similar.

Finally, a teacher’s general satisfaction survey is conducted after the end of the first annual cycle to also evaluate the opinions of the teaching staff. Results of this survey have remained constant over the five editions, with only minimal differences, with a global score of above 8.5 out of 10 (Fig. 4b). The lowest score was related to accessibility of the course’s LMS technological platform (3.1). Several teachers are University professors who are familiar with the use of the LMS in other degrees. However, external teachers face more difficulties in using all its features. It is worth noting that a teacher acting as web manager coordinates transversal activities such as the standardization of the grade book, or the organization of forums, as well as the correct standardization of the material and resource folders, which frees the faculty from these tasks.

### 4.4 Skills acquired by the students by the end of the course are used in their current jobs

**Fig. 5.**
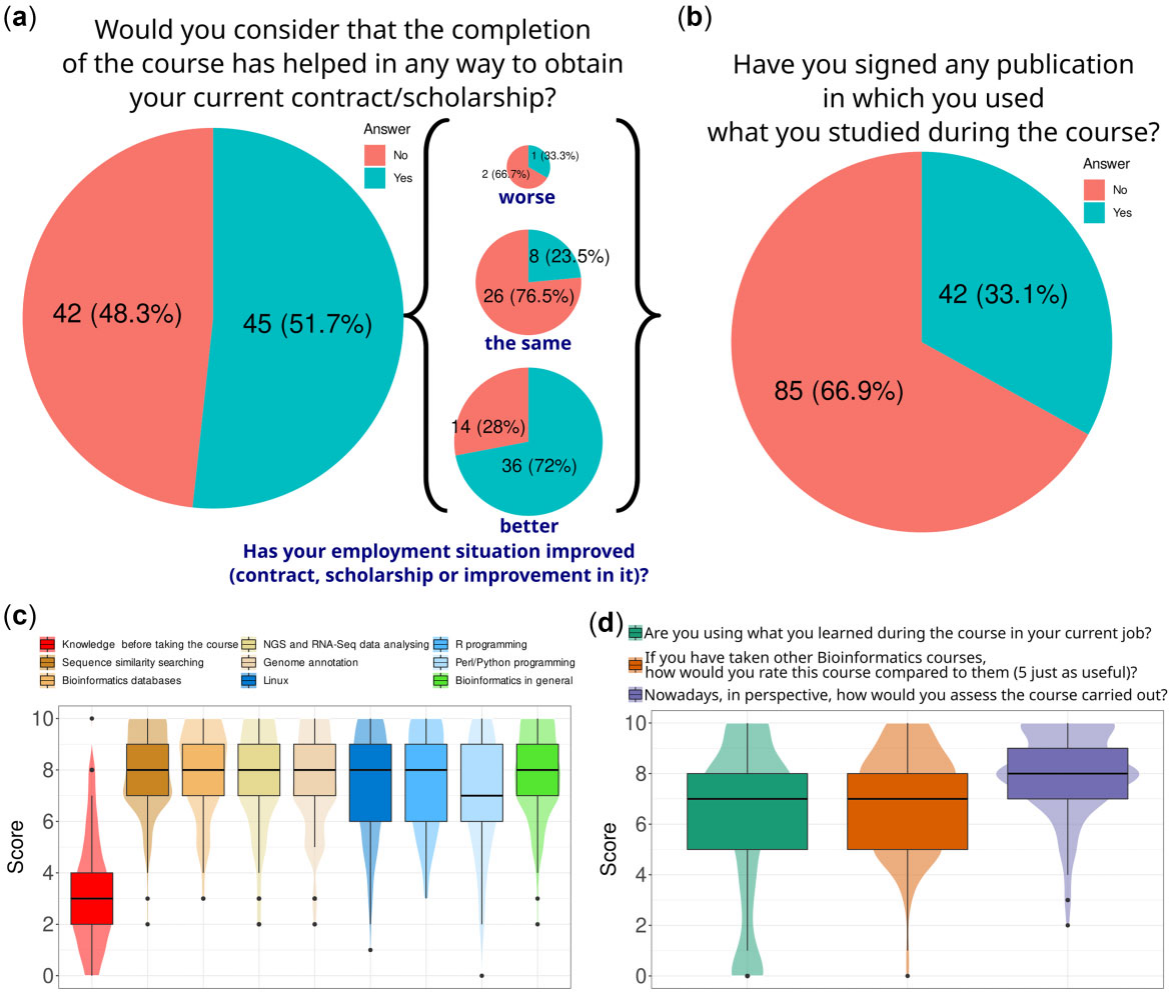
Survey about acquired skills. (**a**) Results of the question as to whether completing the course helped to obtain their current status, which could be answered as yes or no. In brackets, the proportion of students who answered this question distinguished per answer given to the question about whether their status had now improved or not improved. The pie sizes are proportional to the number of students in each group. This number, together with the percent, is shown inside the pies. (**b**) Proportion according to the answer to the question about whether they used what they learnt in a publication. (**c**) Scores given to their starting and final knowledge of bioinformatics. (**d**) Results for the three final questions related to the use of learned availabilities, the comparison with other courses, and the final score that students currently give to the course. In both (c) and (d), the scale ranges from 0 (strongly disagree) to 10 (strongly agree)

The aim of these kinds of courses is training in bioinformatics skills. To measure skill acquisition, a last survey was performed at the end of the course’s fifth edition. We asked all alumni of the 5 editions to fill in this survey, and 127 out of 280 responded. The first question was whether they considered that completing the course helped in any way to obtain their current professional status. Results were differentiated according to whether this status had improved, worsened or remained the same. Around 51% of students answered that the completion of the course had helped to obtain their current status, and 41% of them answered that it had been useful to improve this status (i.e. 36 out the 87 students who answered to this question) (Fig. 5a). One-third of the students also answered that they had used what they had learned in the course in a publication (Fig. 5b). The latter reflects that training competencies are acquired and used to improve their performance.

Furthermore, we sought to measure the competencies acquired in specific practical subjects, comparing them to the students’ initial level of knowledge. Thus, though the majority of students thought that they started from around a level of 3 out of 10 (Fig. 5c), they reached a level that was above seven in subjects such as sequence similarity, databases and next-generation sequencing (NGS) and RNA-Seq analysis, or programming skills in Linux, R and Perl/Python. All of them presented a median value of 8, with only Perl/Python dropping to a level of 7. Therefore, based on students’ answers, the starting median of 3 rose to 8 in ‘bioinformatics in general’.

We finished with three additional questions that further evaluate the course’s success. Students were asked for the use of acquired knowledge and competences in their current job, the comparison of the finished course to others, and the currently perceived value of the course. Again, most students commented that they are currently using what they learned during the course (Fig. 5d). Remarkably, 75% of students thought that this course was better than others they had taken, with only 9 students thinking that it was worse. Finally, 1–5 years after having completed the course, most of the students gave it a high score, with only five students giving a score lower than 5.

## 5 Discussion and future directions

We presented here the structure and results of a modular online bioinformatics course with two, 1-year long blocks requiring part-time dedication. This learning format offers flexibility. Students with heterogeneous profiles from Europe and Latin America have enrolled in the course over the initial five editions. From the beginning, we believed that the learning strategy should be based on practical training and close student monitoring, which implies a greater need for resources and dedication from both teachers and students. However, such a choice has proven to lead to positive results, with a current completion rate of around 84% (Fig. 2c), and high student satisfaction, together with the notable acquisition of key competences (Fig. 5).

In this context, as recommended in recent literature, we have moved away from focusing on teacher-to-student knowledge transmission, in order to maximize results (Lee and Choi, 2011). The course uses learning communities to foster connections among students and teachers, and it creates a place where collaboration is key to knowledge-creation. The subject contents do not rest mainly on written materials and videos, but they are collaboratively completed in the weekly discussion forums in which both students and teachers participate. This active learning model also provides positive results in face-to-face courses (Deslauriers *et al.*, 2019; Ehrlick and Slotta, 2018).

A key factor influencing the results of an online course is early dropout (Hew and Cheung, 2014; Levy, 2007), and even more so when the course has a long duration. This problem is specific to online courses, since traditional ones keep students engaged through the presence in the classroom and peer interaction (Luo, 2014). In relation to the underlying reasons, we showed that close monitoring throughout the course and contacts among students via weekly and evaluable discussion forums could contribute to significantly reducing dropout and keeping the students engaged. Specifically, forums support the creation of collaborative communities that students feel they belong to. Every year, some students complain about the weight of the grades given to forums, but the results and surveys show that they are in fact key tools to keep students engaged in this long-term programme. In addition, students’ perceptions must be considered with caution (Islam, 2013). It was shown, for example, that both teachers and students resisted active teaching strategies and preferred traditional methods, despite better results in acquired competencies (Deslauriers *et al.*, 2019). Nevertheless, keeping up the activity in long-time e-learning courses is a challenge that requires teachers’ active participation (Roddy *et al.*, 2017). We showed that the differentiation of teaching roles, including mentors that monitor small groups and provide rapid-response support to students, can make a major contribution to improving the performance of individual learners. The intended outcome of this mentoring strategy is to reduce the impact of any negative critical incidents on the students’ perception, which has been pointed as a major factor in student dropout (Lin, 2011). Another benefit of close mentoring is to avoid the pernicious effect of procrastination, a notable factor that affects learning outcomes (Goda *et al.*, 2015).

It is not easy to find examples of bioinformatic courses similar to the one presented here in which this parameter has been studied. Our course has shown an average dropout rate of 26%, which in the last edition was reduced to only 16% (Fig. 2c). This contrasts with MOOCs rates, that average 87% (Jordan, 2015), or with specific studies such as a recently published one of computer programming MOOCs that reported 45–60% (Rõõm *et al.*, 2021). A fully online bioinformatics course from which we can extract its dropout rate is the first S-star Trial Bioinformatics online course, a modular course offered by the S* Life Science Informatics Alliance which involved six institutions worldwide (Lim *et al.*, 2003). This programme reached a 41% dropout rate with an initial number of students of 150, who presented similar profiles to that of our students. Although it should be noted that this course took place from October to November 2001, so the comparison is not entirely balanced due to the technological and attitudinal changes that have taken place since then. The physical presence in a course of these characteristics is something that helps to improve the retention of students in the programme. Several African institutions belonging to H3ABioNet have been organizing bioinformatics courses for years, mainly blended or hybrid learning courses (Gurwitz *et al.*, 2017), that combine online and face-to-face teaching. If we take the detailed results offered in 2017 by the University of Khartoum on one of these courses, results that can be considered as a reference of good practices for this type of courses, we can see that they obtained a dropout rate of 26% (Ahmed *et al.*, 2020). This value is equal to the average obtained by our course over five editions, which can be considered a very high value and rarely obtained in e-learning (Levy, 2007).

One of the reasons for obtaining dropout rates similar to those of other courses that include face-to-face attendance, we believe, may be the uniform structure of our course, as well as its homogeneous scheme of work, together with the monitoring and evaluation system of the discussion forums. Although flexibility is often perceived as one of the advantages of online learning, we believe that the proposed weekly schedule has been a crucial feature to retain students and avoid procrastination. Indeed, it establishes the students’ calendar and activities, and the tasks’ deadlines are near in time.

Another important feature of any university course is quality control, consisting in a regular evaluation in order to ensure continuous improvement (Westerlaken *et al.*, 2019). It is usually based on students’ suggestions, but also on teachers’ perceptions and the specific improvement areas they detect during the course. It ensures that the course quality can only increase, since new editions do not reproduce the same errors as those of previous years. In this process, it is important to reach a minimum rate of participation; courses sometimes provide rewards or incentives to promote this participation (Hessler *et al.*, 2018). However, we have shown that when students are engaged, their participation can be sufficient, even when they receive a survey each month (Fig. 4a). In fact, we believe that this quality management system has also helped to decrease the dropout rate of the course, since it allows us to detect weak points year by year, not only at the level of the entire course but also subject by subject.

The course has acquired a high reputation in Spain during its 5 years of existence, due to the transmission by word of mouth from former students, and its relatively low cost (750–795€ per year) and scholarship programme. High fees can sometimes explain low dropout rates in postgraduate courses, but the low 16% of abandonment in our course cannot be attributed to high tuition costs. Conversely, it can be explained by the student perception about what they have learned during the year of duration. In forthcoming editions of the course, we want to improve the evaluation of the students’ gain of bioinformatics competences. To this end, we want to carry out a survey on the first day of class, the results of which we will compare with a repeat of the same survey at the end of the course.

Finally, scalability is often seen as an intrinsic characteristic of online learning. It is sometimes assumed that once the instructional design is deployed, it can be applied to any number of students. This course has become very popular as shown by the increase in the number of applications. This number now usually reaches more than 100 applications in each edition. This was also achieved during the years of the COVID-19 pandemic (Sanche *et al.*, 2020), with 103 in the edition 2020–2021 and 87 in the edition 2021–2022. Reaching more than 100 applications meant that from the fourth edition onwards we increased the number of students admitted annually from 60 to 80. This was a challenge due to the high number of students working and discussing over forums, but it did not compromise any of the aspects of the course, since we added a new tutor per subject to maintain the teaching model and the mentor/student ratio. In fact, grades and performance rates obtained during the last two years have been higher (Fig. 2). The explanation may lie in a combination of our continuous improvement system and our current admission programme that prioritizes profiles with a more complete curriculum over recent graduates. We do believe, however, that it constitutes new evidence that the presented model works for this kind of teaching, even when the number of students is high, provided that the mentor/student ratio is maintained.

## 6 Conclusions and future ideas

At present, we offer a part-time 1-year course, which is in great demand and backed by word of mouth, and which is also giving good results and obtaining very positive feedback from students. The second year optional course has a more limited number of admissions, as one-third of the time is devoted to a master’s thesis, consisting of a practical training project, and the number of proposals is limited. Moreover, we have detected a growing interest on the part of new postgraduate students who wish to direct their studies toward bioinformatics. However, their access to our current course is hampered by their limited professional experience. Consequently, we have recently made a request to establish an official Master’s degree in bioinformatics to manage this demand, based on our current non-official Master’s degree. For the time being, we are keeping the programme’s first module as a lifelong learning programme offered to professionals seeking to acquire bioinformatics skills for their daily work.

## Supplementary Material

vbac031_Supplementary_Data
