# Supplementary material for: E-learning strategies from a bioinformatics postgraduate programme to improve student engagement and completion rate
Source: Bioinform Adv. 2022 May 10;2(1):vbac031. doi: 10.1093/bioadv/vbac031 (PMC9710613; doi:10.1093/bioadv/vbac031)
Supplement: vbac031_Supplementary_Data [file vbac031_supplementary_data.zip › suppl1.docx]

**Subject _______**

**Improvement plan evaluation. Course _________**

| Action taken | Compliance indicator_1_ | Compliance indicator evaluation_3_ | Performance indicator_2_ | Performance indicator evaluation_4_ |
| --- | --- | --- | --- | --- |
| Action 1.- Improvement of the forums' organization. Commitment to organize the number of open discussions and participation in them. Increase the number of questions and topics for discussion (some article or interesting aspect). Activate the different debates progressively. Create separate debates for individual questions. In this way we can separate contributions to discussion topics from contributions to help classmates. Prevent students from opening redundant debates. | Increase in the number of debates opened by the faculty (12 in the past academic year). Sequential appearance of the debates. | 22 debates opened by the teaching staff. Of these, 15 at the beginning of the week and 7 sequentially at different times. | Improving student performance in debates.  Student’s participation in debates.  Number of complaints. | Average score in forums improved from 114/400 to 144/400.  Percentage of students not participating in forums reduced from 30% to 16.5%.  Improvement from 9 negative comments and one positive to 2 negative and 3 positive comments |
| Action 2.- |  |  |  |  |
| Action 3.- |  |  |  |  |

**Global analysis_5_:**

**Improvement plan course ________**

**Analysis of the evidence_6_**:

**Improvement plan_7_**

| Action to take | Compliance indicator_1_ | Performance indicator_2_ |
| --- | --- | --- |
| Action 1 |  |  |
| Action 2 |  |  |
| Action 3 |  |  |

**Notes**

**1.- Compliance indicators: List the objective evidence that will make it possible to verify whether the improvement action has been implemented (regardless of its effectiveness). As far as possible, they should allow the quantification of compliance.**

**2.- Performance indicator: List those indicators that will allow to verify the corrective effect of the improvement action. As far as possible, they should be quantitative indicators that allow to assess the degree of effectiveness by comparing values of the current course with the following one. For quantitative indicators it is convenient to identify the value of the proposed indicator corresponding to the reference course.**

**3.- Compliance indicator evaluation. Provide evidence of compliance with the improvement action during the current academic year. The evidence must be based on the compliance indicators established the previous year.**

**4.- Performance indicator evaluation. Provide evidence on the corrective result of the improvement action. They must be based on the indicators defined the previous year for the improvement action.**

**5.- Global analysis. An evaluation will be made on the degree of compliance and effectiveness of the measures adopted. As far as possible, the lack of compliance or effect of the measure will be justified. The continuation of a measure for the following year may be proposed.**

**6.- Analysis of the evidence. Identify evidence that denote weaknesses or threats related to the subject and that could be improved by the teaching team. The results of the student surveys and the perceptions of the teaching staff can be used for this purpose.**

**7.- Improvement plan: A maximum of three improvement actions will be made to be implemented next year. For a correct evaluation it is very important to define proper compliance and result indicators, as well as to provide the value of the quantitative indicators in this course for comparison with the next course.**
