# Supplementary material for: E-learning strategies from a bioinformatics postgraduate programme to improve student engagement and completion rate
Source: Bioinform Adv. 2022 May 10;2(1):vbac031. doi: 10.1093/bioadv/vbac031 (PMC9710613; doi:10.1093/bioadv/vbac031)
Supplement: vbac031_Supplementary_Data [file vbac031_supplementary_data.zip › suppl2.docx]

1. CONTENTS
   1. The contents of the course have been interesting and according to my expectations.
   2. The contents and activities in the subject were well structured and I had no problem understanding what I had to do at each moment.
   3. The materials are clear, quality and have helped me in my learning.
2. TUTORING
   1. My tutor has attended me with the necessary diligence.
   2. My tutor has resolved my doubts and has helped me to understand the subject.
   3. My tutor has made correct suggestions to me, without asking for it, depending on my difficulties in the subject.
3. FORUMS AND LEARNING COMMUNITY
   1. The forums have been useful for my learning.
   2. I consider that the tutors have moderated the forums in an adequate way, providing relevant information and correcting errors.
   3. I have learned from my peers.
4. USEFULNESS
   1. I consider that I have acquired skills that enable me to work in the field of the subject.
5. PERSONAL EFFORT
   1. I consider that my attitude as a student has been good.
   2. In theory I should have dedicated 25 hours a week of work to the subject. In fact, I have dedicated to it (choose between 1: much less, 2: less, 3: more or less, 4: more, 5: much more).
6. GLOBAL ASSESSMENT
   1. Globally rank from 1 (very bad) to 5 (very good) the quality of the subject.
