# Supplementary material for: E-learning strategies from a bioinformatics postgraduate programme to improve student engagement and completion rate
Source: Bioinform Adv. 2022 May 10;2(1):vbac031. doi: 10.1093/bioadv/vbac031 (PMC9710613; doi:10.1093/bioadv/vbac031)
Supplement: vbac031_Supplementary_Data [file vbac031_supplementary_data.zip › suppl3.docx]

1. OBJECTIVES AND CONTENTS
   1. The objectives of the course were achieved.
   2. The contents are clear and adapted to the training needs to be covered.
   3. The duration of the course was adequate in relation to the contents.
2. METHODOLOGY
   1. The methodology and the planning were appropriate according to the objectives and contents of the course.
   2. Following the online course represented a greater personal effort compared to a traditional face-to-face or distance course.
3. TEACHING
   1. The teaching staff has adequate knowledge about the contents of the course
   2. Practices, exercises and examples have favored learning.
   3. The teaching staff responded adequately to the questions proposed.
   4. Global evaluation of teaching.
4. MATERIAL AND DIDACTIC RESOURCES
   1. Accessibility and general operation of the technological platform on which the course has been given.
   2. Ease and level of use of the communication tools (mail, forum, board...) with the tutor and the rest of the participants.
   3. Additional teaching materials and resources (documentation, links to websites, videos, glossary, etc.) were sufficient.
   4. Effectiveness of the means at your disposal to resolve doubts and technical problems.
   5. Presentation and organization of contents.
   6. Activities and case studies.
   7. Self-correction exercises.
5. USEFULNESS
   1. The lessons received are useful for your professional development.
   2. The lessons received are useful for your personal training.
6. UNIVERSITY MANAGEMENT
   1. Evaluate the University management.
7. SUMMARY
   1. The course deserves an overall rating of.
