# Supplementary material for: E-learning strategies from a bioinformatics postgraduate programme to improve student engagement and completion rate
Source: Bioinform Adv. 2022 May 10;2(1):vbac031. doi: 10.1093/bioadv/vbac031 (PMC9710613; doi:10.1093/bioadv/vbac031)
Supplement: vbac031_Supplementary_Data [file vbac031_supplementary_data.zip › suppl4.docx]

1. OBJECTIVES AND CONTENTS
   1. The objectives of the module were achieved.
   2. Assess the degree of fulfillment of the programme planned for the course or module.
   3. The duration of the module was adequate in relation to the contents.
2. METHODOLOGY
   1. The methodology and planning were appropriated according to the objectives and contents of the module.
   2. The activities and the exercises have allowed the students to understand the theoretical contents.
3. LEARNING RESOURCES
   1. Accessibility and general operation of the technological platform on which the module has been taught.
   2. Evaluate the adequacy of the didactic resources (forums, chat, e-mail, etc.).
   3. Assess the adequacy of activities, case studies and self-assessment tests
4. TUTORING
   1. The contribution of the tutor has been important in the training process.
5. UNIVERSITY MANAGEMENT
   1. Evaluate the management by the University.
6. STUDENTS
   1. Evaluate the performance achieved by the students with respect to their module.
   2. Assess the satisfaction perceived in the student with regard to the course.
7. SUMMARY
   1. The module deserves an overall assessment of.
