# Supplementary material for: E-learning strategies from a bioinformatics postgraduate programme to improve student engagement and completion rate
Source: Bioinform Adv. 2022 May 10;2(1):vbac031. doi: 10.1093/bioadv/vbac031 (PMC9710613; doi:10.1093/bioadv/vbac031)
Supplement: vbac031_Supplementary_Data [file vbac031_supplementary_data.zip › suppl7.docx]

**FIRST YEAR**

**MODULE I: BASICS AND FUNDAMENTALS OF BIOINFORMATICS. 18 ECTS credits**

**Subject 1: Biological basis of bioinformatics.**

1. Structure and study of genomes.

2. Genome functions I. Expression of information.

3. Genome functions II. Replication and inheritance.

4. Genomes’ evolution.

5. Omics.

6. Functional Genomics and genomes projects.

**Subject 2: Molecular sequence management.**

1. Sequence similarity searches.

2. Sequence databases.

3. Omics databases.

**Subject 3: Introduction to the R programming language.**

1. Introduction to programming.

2. Introduction to R.

3. Command line R for statistical analysis.

4. Graphical representation of data in R.

5. Programming in R.

**Subject 4: Linux Operating System and High-Performance Computing.**

1. Installation of a Linux operating system.

2. Files and directory trees.

3. Linux terminal commands.

4. Input/output redirection (piping).

5. Using Blast from the Linux command terminal.

6. Accessing and working remotely to supercomputing clusters.

**Subject 5: Introduction to programming to solve biological problems.**

1. Introduction to Python.

2. Interactive Python session.

3. Creating and executing programs.

4. Operators.

5. Strings, lists, tuples and dictionaries.

6. Regular expressions.

7. Standard input: input() and Standard output print().

8. File operations.

**MODULE II: LARGE-SCALE ANALYSIS OF BIOLOGICAL DATA. 12 ECTS credits**

**Subject 6: Genomic data analysis: Next Generation Sequencing (NGS).**

1. Introduction to Next Generation Sequencing (NGS) technologies.

2. Quality analysis and sequence filtering.

3. Sequence mapping: mapping, visualization and quality analysis tools.

4. Genomic variant identification and annotation.

5. De novo genome assembly.

**Subject 7: Analysis of gene expression and regulation data.**

1. Gene expression analysis by RNA-Seq.

2. Gene expression analysis by scRNA-Seq.

2. De novo assembly of transcriptomes.

3. Regulatory data analysis: ChIP-Seq.

**Subject 8: Structural and functional annotation of genomes.**

1. Introduction to gene search and gene detection in prokaryotic organisms.

2. Gene prediction in prokaryotic organisms.

3. Gene prediction in eukaryotic organisms.

4. Introduction to the evaluation of structural predictions of genes.

5. Obtaining annotations.

6. Mass annotation.

**SECOND YEAR**

**MODULE III: ADVANCED BIOINFORMATICS. 20 ECTS credits**

**Subject 9: Design of experiments.**

1. Basic statistics

2. One-factor designs

3. Crossover designs

4. Hierarchical designs

**Subject 10: Advanced computer programming.**

1. Basic concepts of Python

2. Orderly programming with Python

3. Functions

4. Introduction to Modules

5. Code Documentation (Docstrings)

6. Errors and Exceptions

7. Regular expressions. Module re

8. Execution of external commands

9. Statistical calculation

10. Creation of modules

**Subject 11: Protein structure analysis.**

1. Introduction to protein structures

2. 3D structure modeling

3. Structure Comparison

4. 3D structure analysis

**Subject 12: Gene networks.**

1. Introduction to the prediction, modeling and use of molecular interaction networks.

2. Computational and experimental methods for systemic prediction of gene interactions.

3. Similarity and distance metrics between interacting gene pairs.

4. Gene Ontology and semantic similarity networks.

5. Validation and comparison of prediction methods. ROC curves.

6. Integration of prediction methods.

7. Clustering and functional analysis in molecular interaction networks.

**Subject 13: Data Science and Big Data.**

1. Introduction to Data Science.

2. Data preprocessing: Cleaning, editing, attribute selection.

3. Regression.

4. Classification.

5. Clustering.

6. Association rules.

7. Introduction to Big Data.

8. Applications: A real case.

**MODULE IV: MASTER'S THESIS. 10 ECTS credits**
