# Supplementary material for: E-learning strategies from a bioinformatics postgraduate programme to improve student engagement and completion rate
Source: Bioinform Adv. 2022 May 10;2(1):vbac031. doi: 10.1093/bioadv/vbac031 (PMC9710613; doi:10.1093/bioadv/vbac031)
Supplement: vbac031_Supplementary_Data [file vbac031_supplementary_data.zip › Suppl. Fig. S1.pdf]

# BBB

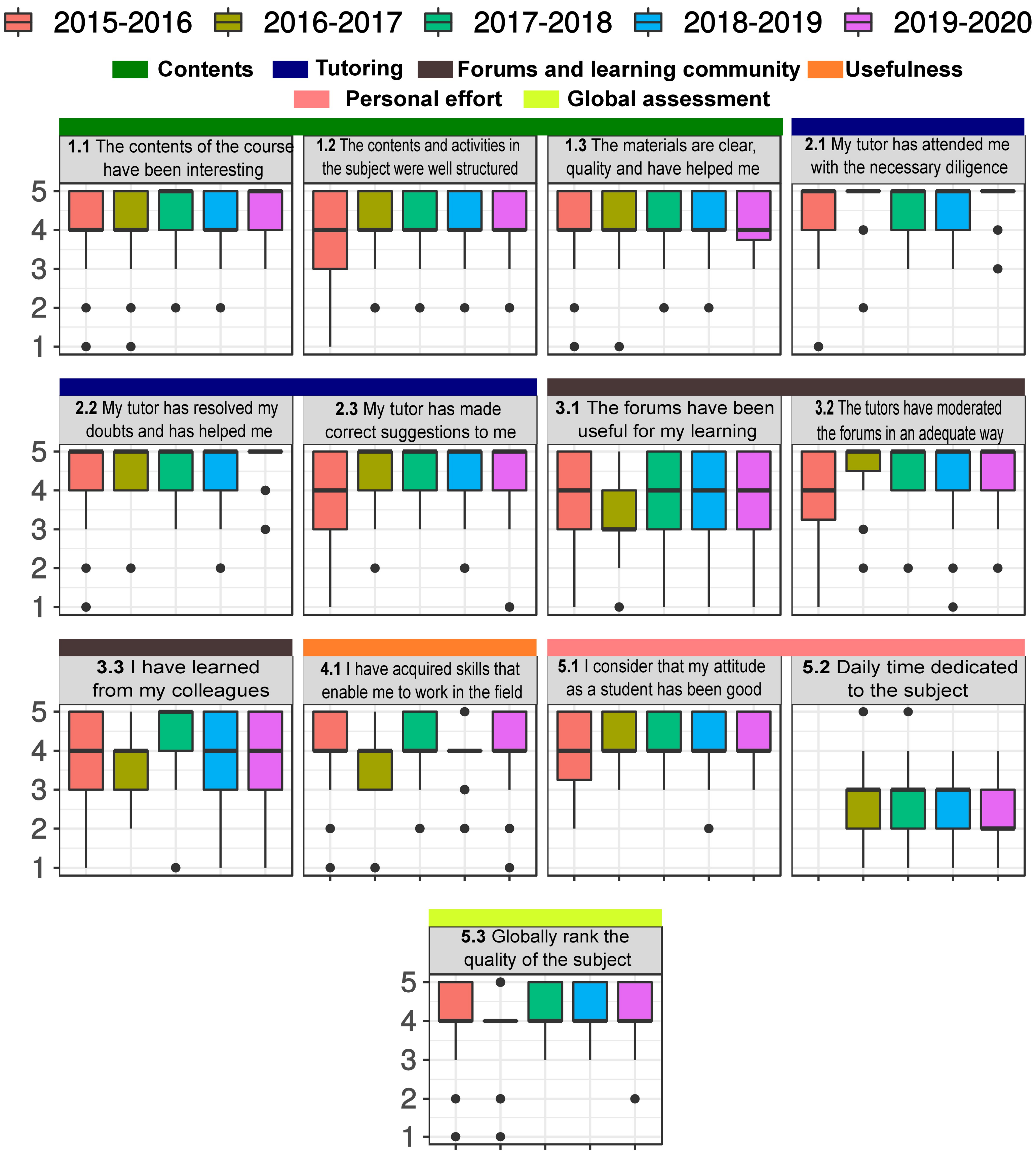

2015-2016
2016-2017
2017-2018
2018-2019
2019-2020
Contents
Tutoring
Forums and learning community
Usefulness
Personal effort
Global assessment
1.1 The contents of the course have been interesting
1.2 The contents and activities in the subject were well structured
1.3 The materials are clear, quality and have helped me
2.1 My tutor has attended me with the necessary diligence
2.2 My tutor has resolved my doubts and has helped me
2.3 My tutor has made correct suggestions to me
3.1 The forums have been useful for my learning
3.2 The tutors have moderated the forums in an adequate way
3.3 I have learned from my colleagues
4.1 I have acquired skills that enable me to work in the field
5.1 I consider that my attitude as a student has been good
5.2 Daily time dedicated to the subject
5.3 Globally rank the quality of the subject
1
2
3
4
5

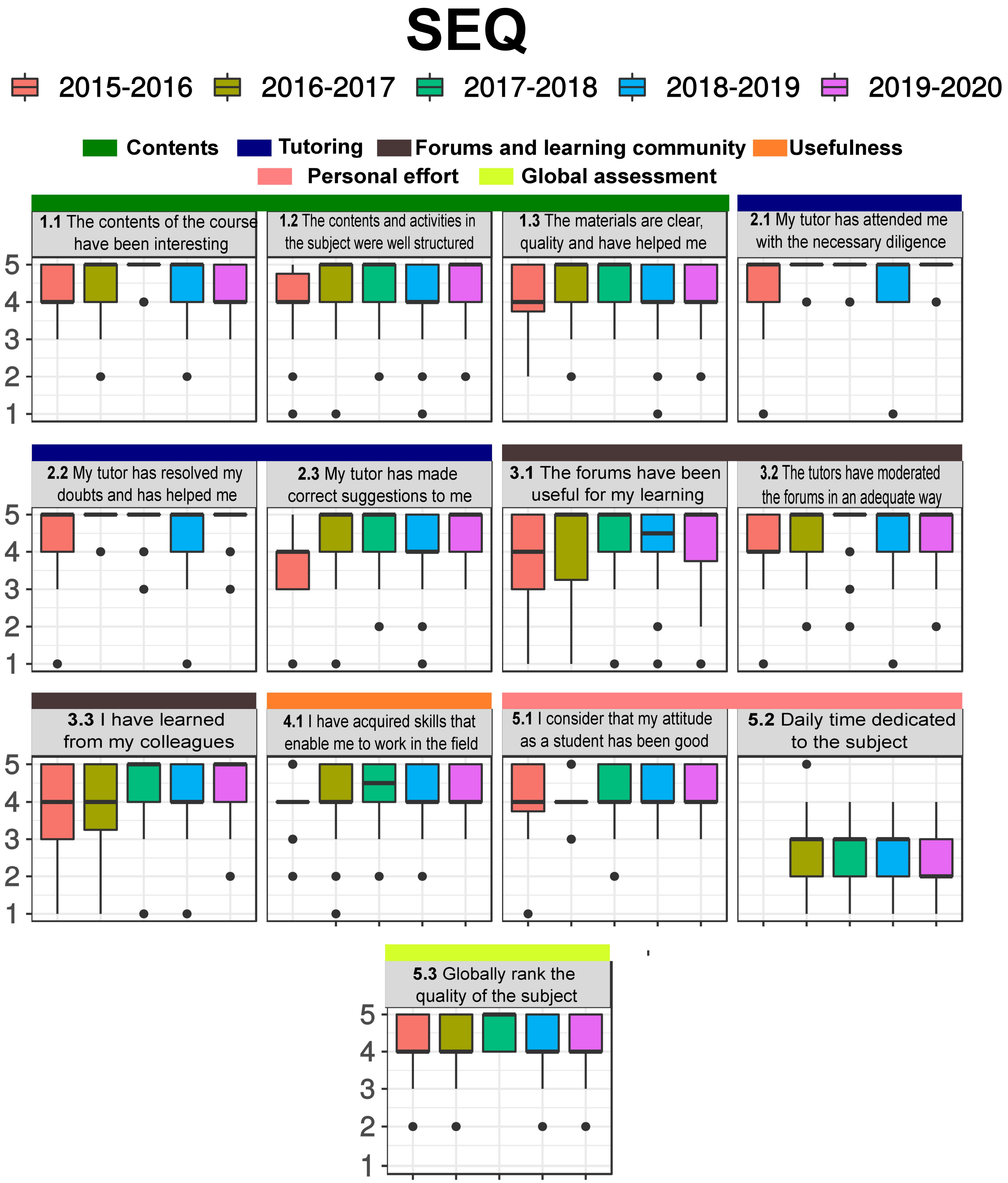
SEQ
2015-2016
2016-2017
2017-2018
2018-2019
2019-2020
Contents
Tutoring
Forums and learning community
Usefulness
Personal effort
Global assessment
1.1 The contents of the course have been interesting
1.2 The contents and activities in the subject were well structured
1.3 The materials are clear, quality and have helped me
2.1 My tutor has attended me with the necessary diligence
2.2 My tutor has resolved my doubts and has helped me
2.3 My tutor has made correct suggestions to me
3.1 The forums have been useful for my learning
3.2 The tutors have moderated the forums in an adequate way
3.3 I have learned from my colleagues
4.1 I have acquired skills that enable me to work in the field
5.1 I consider that my attitude as a student has been good
5.2 Daily time dedicated to the subject
5.3 Globally rank the quality of the subject
1
2
3
4
5

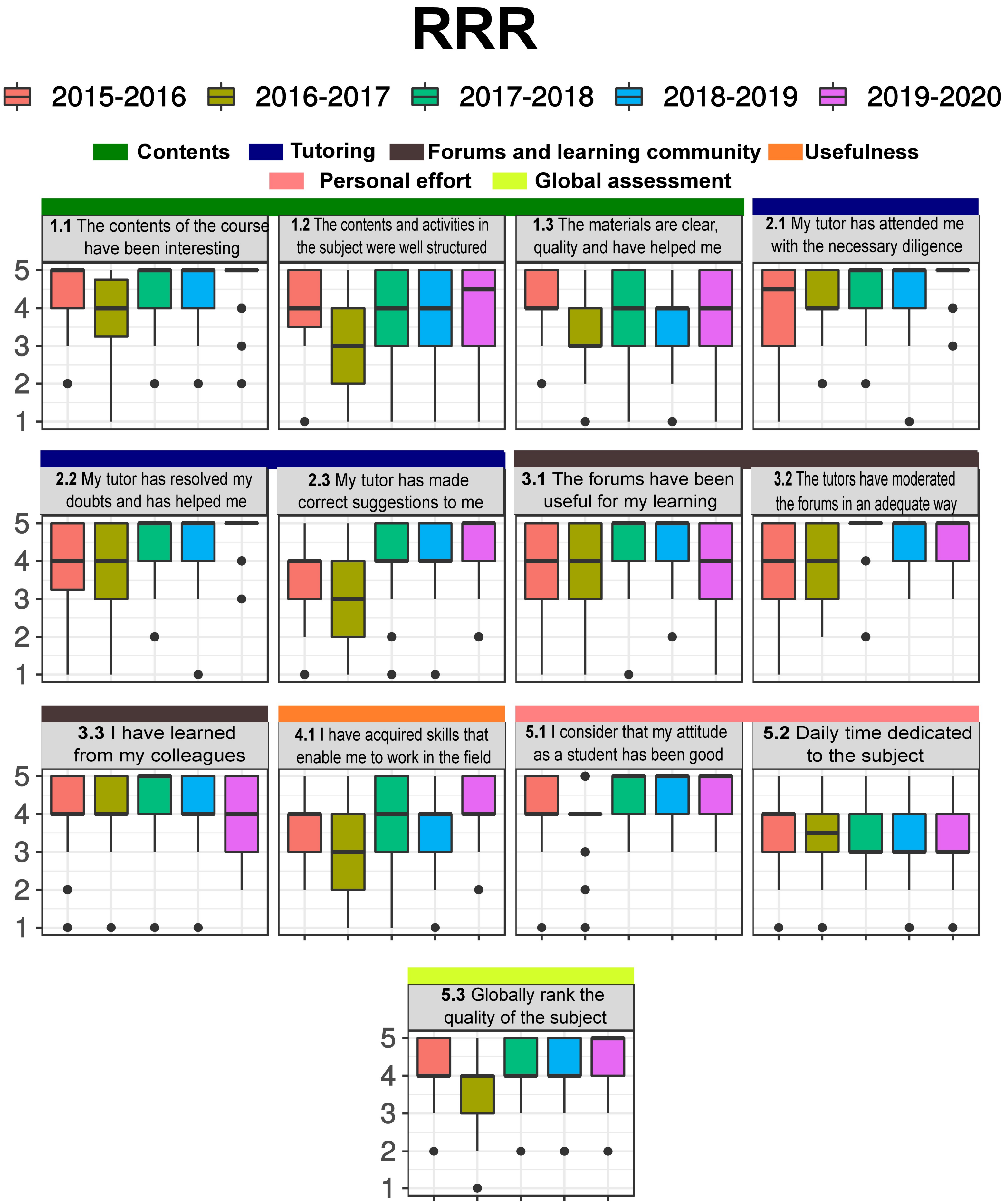

RRR
2015-2016
2016-2017
2017-2018
2018-2019
2019-2020
Contents
Tutoring
Forums and learning community
Usefulness
Personal effort
Global assessment
1.1 The contents of the course have been interesting
1.2 The contents and activities in the subject were well structured
1.3 The materials are clear, quality and have helped me
2.1 My tutor has attended me with the necessary diligence
2.2 My tutor has resolved my doubts and has helped me
2.3 My tutor has made correct suggestions to me
3.1 The forums have been useful for my learning
3.2 The tutors have moderated the forums in an adequate way
3.3 I have learned from my colleagues
4.1 I have acquired skills that enable me to work in the field
5.1 I consider that my attitude as a student has been good
5.2 Daily time dedicated to the subject
5.3 Globally rank the quality of the subject
1
2
3
4
5

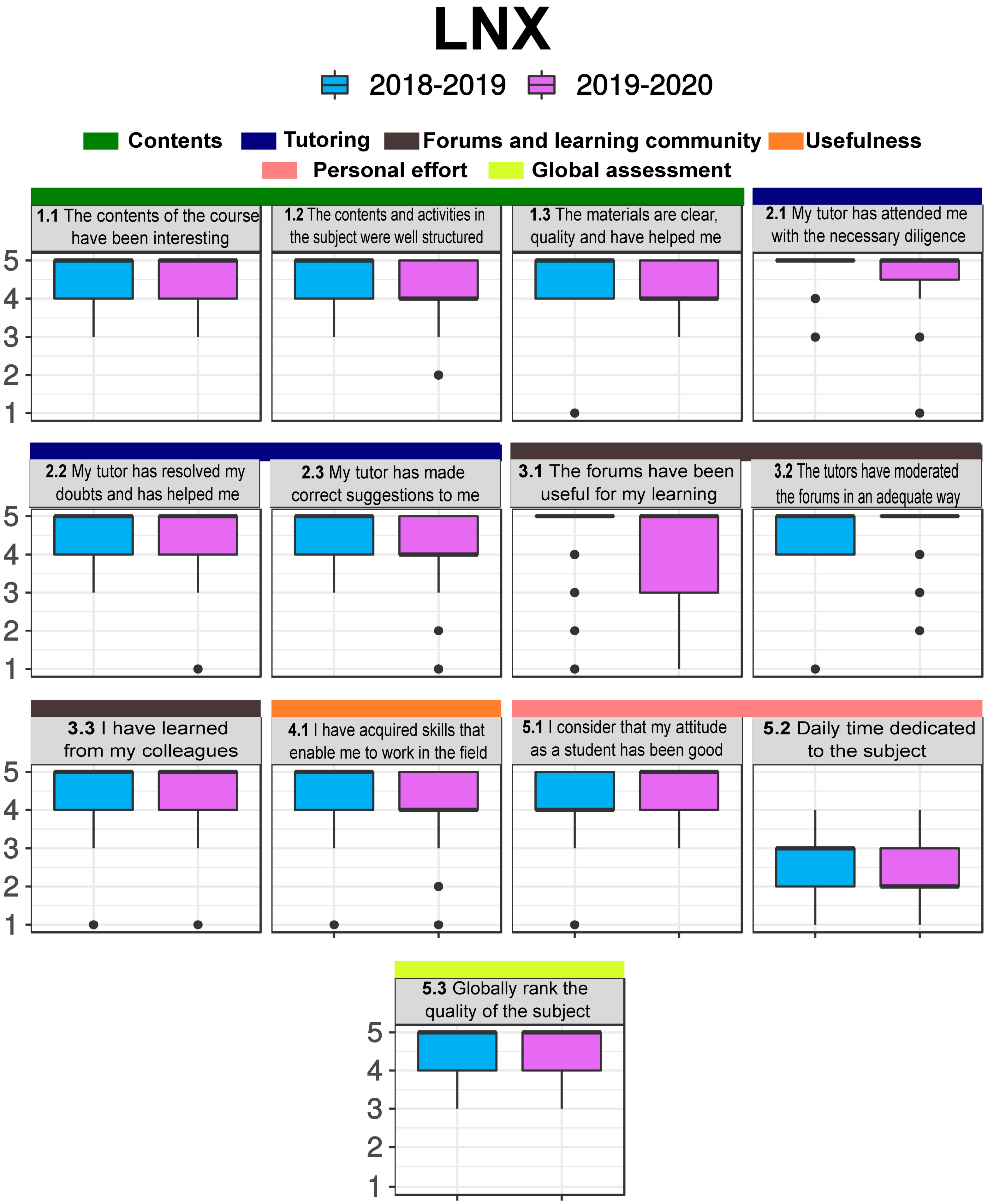
LNX
2018-2019
2019-2020
Contents
Tutoring
Forums and learning community
Usefulness
Personal effort
Global assessment
1.1 The contents of the course have been interesting
1.2 The contents and activities in the subject were well structured
1.3 The materials are clear, quality and have helped me
2.1 My tutor has attended me with the necessary diligence
2.2 My tutor has resolved my doubts and has helped me
2.3 My tutor has made correct suggestions to me
3.1 The forums have been useful for my learning
3.2 The tutors have moderated the forums in an adequate way
3.3 I have learned from my colleagues
4.1 I have acquired skills that enable me to work in the field
5.1 I consider that my attitude as a student has been good
5.2 Daily time dedicated to the subject
5.3 Globally rank the quality of the subject
5
4
3
2
1

# PRG

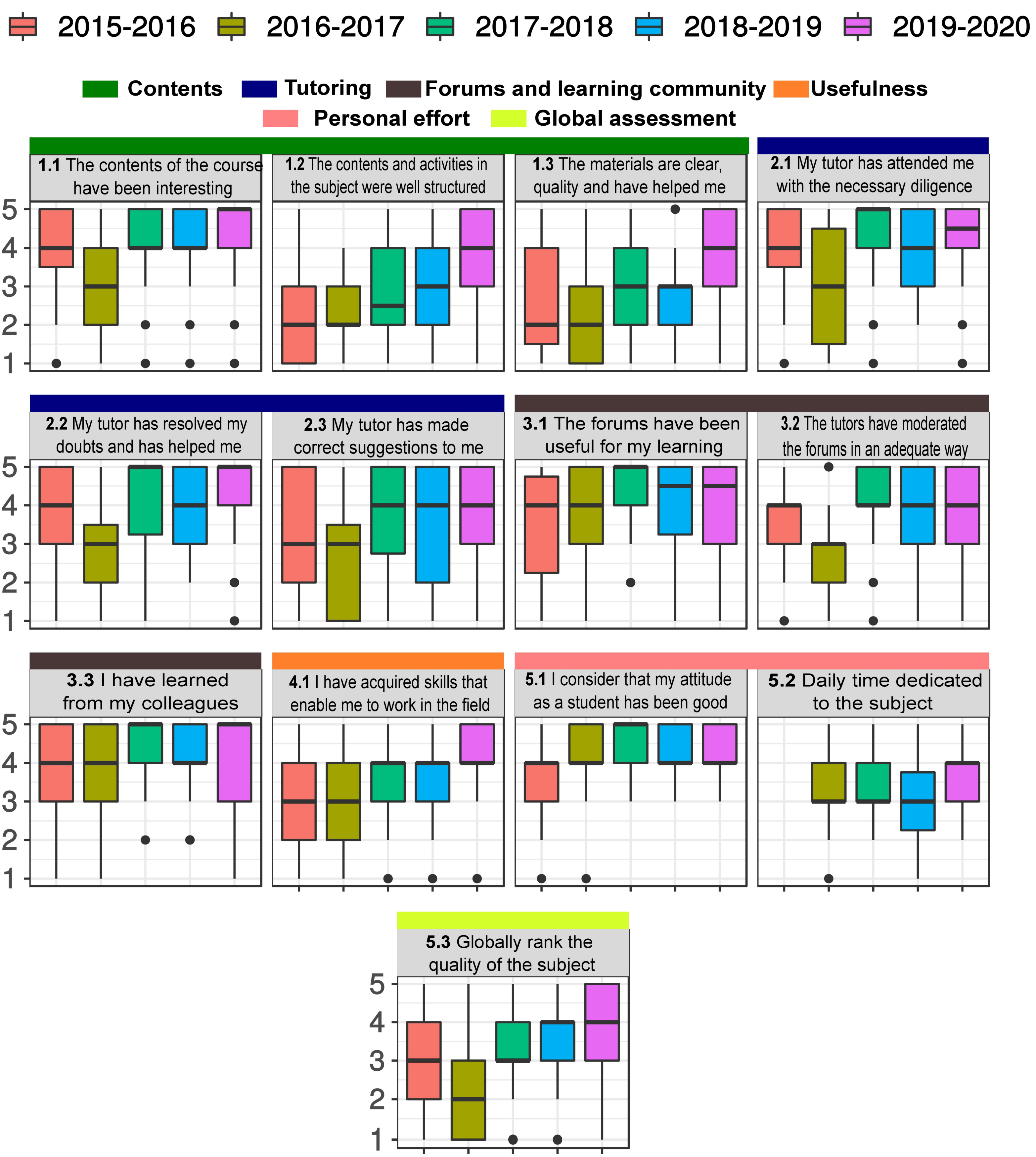
2015-2016
2016-2017
2017-2018
2018-2019
2019-2020
Contents
Tutoring
Forums and learning community
Usefulness
Personal effort
Global assessment
1.1 The contents of the course have been interesting
1.2 The contents and activities in the subject were well structured
1.3 The materials are clear, quality and have helped me
2.1 My tutor has attended me with the necessary diligence
2.2 My tutor has resolved my doubts and has helped me
2.3 My tutor has made correct suggestions to me
3.1 The forums have been useful for my learning
3.2 The tutors have moderated the forums in an adequate way
3.3 I have learned from my colleagues
4.1 I have acquired skills that enable me to work in the field
5.1 I consider that my attitude as a student has been good
5.2 Daily time dedicated to the subject
5.3 Globally rank the quality of the subject
1
2
3
4
5

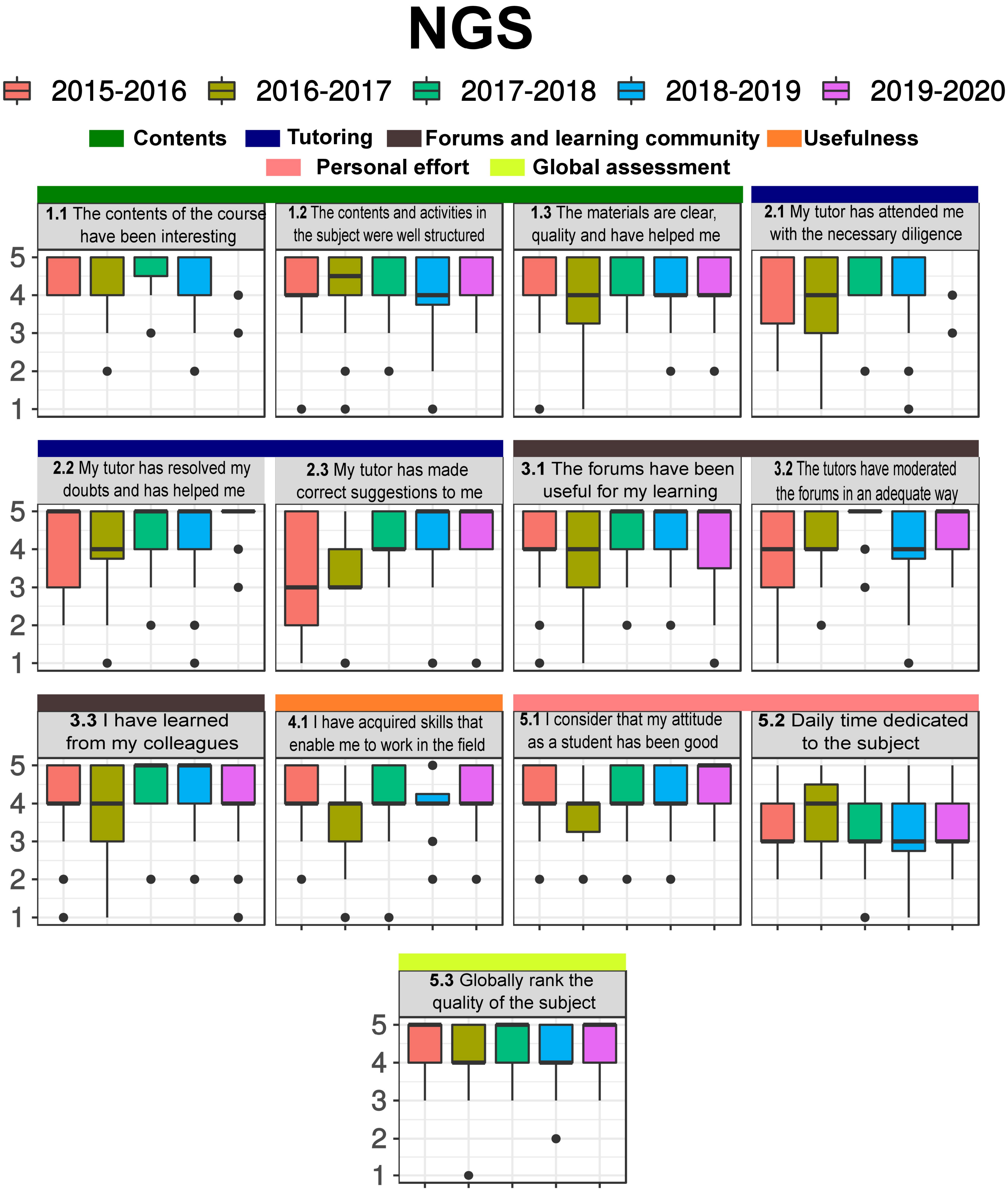
NGS
2015-2016
2016-2017
2017-2018
2018-2019
2019-2020
Contents
Tutoring
Forums and learning community
Usefulness
Personal effort
Global assessment
1.1 The contents of the course have been interesting
1.2 The contents and activities in the subject were well structured
1.3 The materials are clear, quality and have helped me
2.1 My tutor has attended me with the necessary diligence
2.2 My tutor has resolved my doubts and has helped me
2.3 My tutor has made correct suggestions to me
3.1 The forums have been useful for my learning
3.2 The tutors have moderated the forums in an adequate way
3.3 I have learned from my colleagues
4.1 I have acquired skills that enable me to work in the field
5.1 I consider that my attitude as a student has been good
5.2 Daily time dedicated to the subject
5.3 Globally rank the quality of the subject
1
2
3
4
5

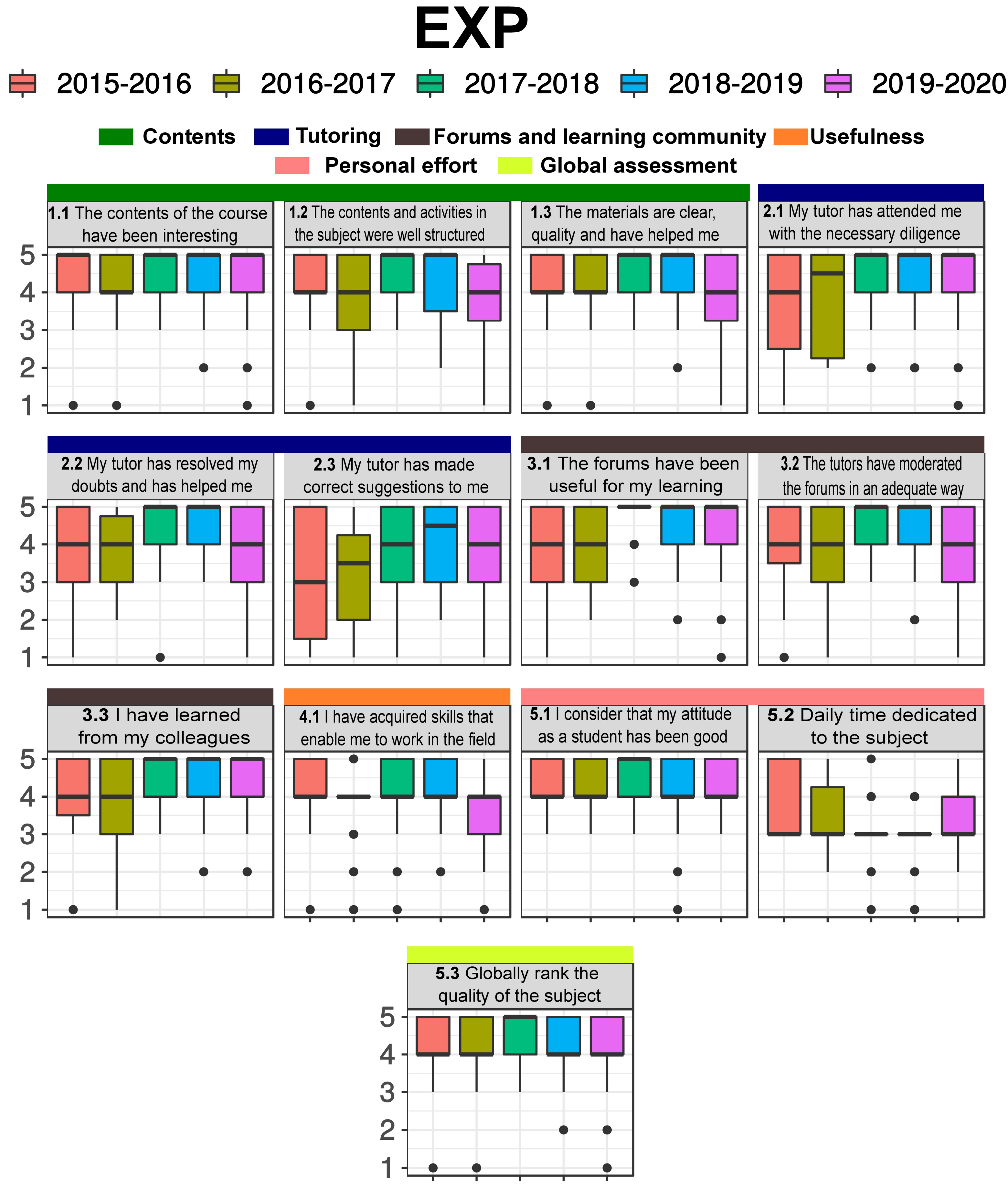

EXP
2015-2016
2016-2017
2017-2018
2018-2019
2019-2020
Contents
Tutoring
Forums and learning community
Usefulness
Personal effort
Global assessment
1.1 The contents of the course have been interesting
1.2 The contents and activities in the subject were well structured
1.3 The materials are clear, quality and have helped me
2.1 My tutor has attended me with the necessary diligence
2.2 My tutor has resolved my doubts and has helped me
2.3 My tutor has made correct suggestions to me
3.1 The forums have been useful for my learning
3.2 The tutors have moderated the forums in an adequate way
3.3 I have learned from my colleagues
4.1 I have acquired skills that enable me to work in the field
5.1 I consider that my attitude as a student has been good
5.2 Daily time dedicated to the subject
5.3 Globally rank the quality of the subject
5
4
3
2
1

# ANT

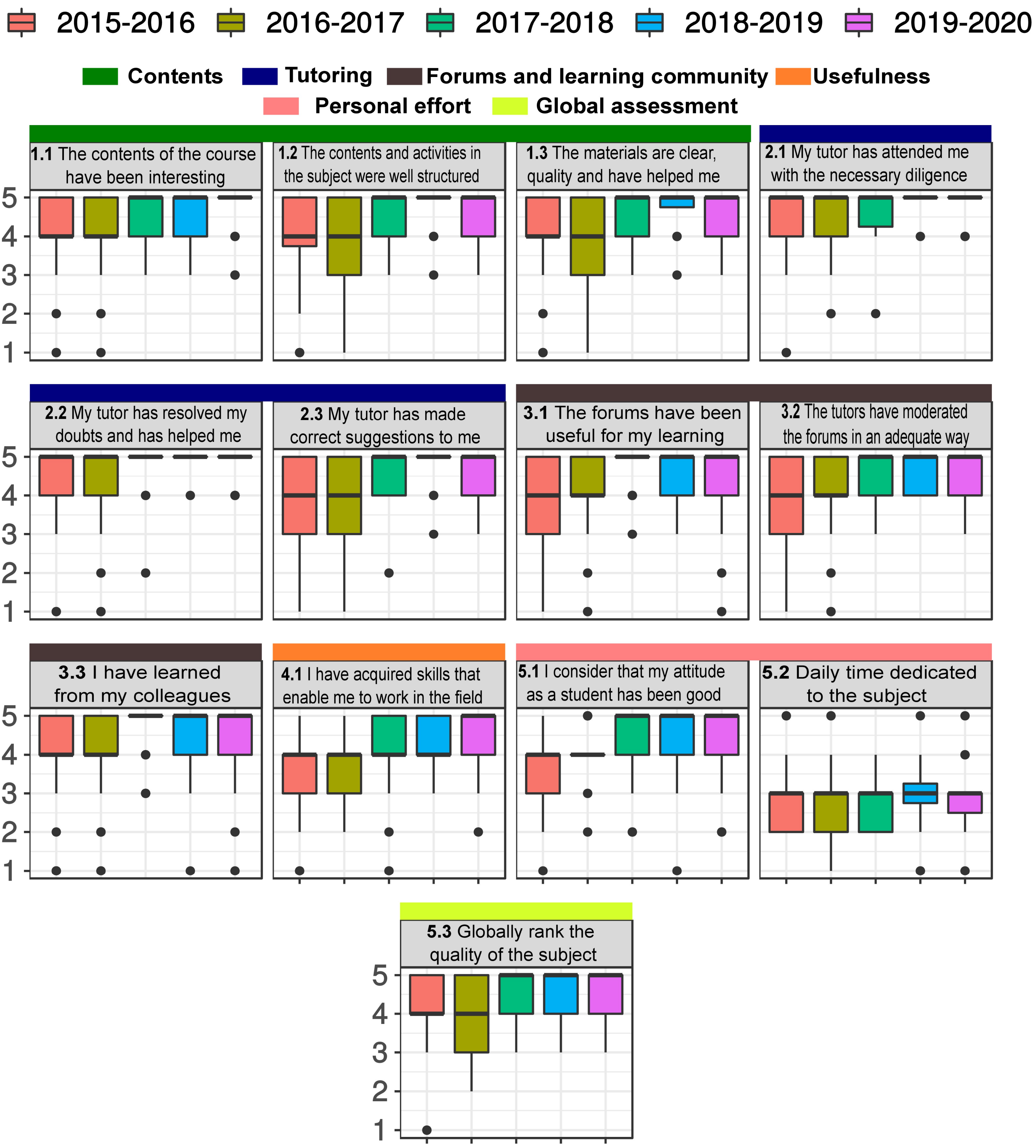

2015-2016
2016-2017
2017-2018
2018-2019
2019-2020
Contents
Tutoring
Forums and learning community
Usefulness
Personal effort
Global assessment
1.1 The contents of the course have been interesting
1.2 The contents and activities in the subject were well structured
1.3 The materials are clear, quality and have helped me
2.1 My tutor has attended me with the necessary diligence
2.2 My tutor has resolved my doubts and has helped me
2.3 My tutor has made correct suggestions to me
3.1 The forums have been useful for my learning
3.2 The tutors have moderated the forums in an adequate way
3.3 I have learned from my colleagues
4.1 I have acquired skills that enable me to work in the field
5.1 I consider that my attitude as a student has been good
5.2 Daily time dedicated to the subject
5.3 Globally rank the quality of the subject
1
2
3
4
5
